# Red Cell Distribution Width as a Predictor of Mortality in Patients With Clinical Sepsis: Experience From a Single Rural Center in Central India

**DOI:** 10.1177/2632010X221075592

**Published:** 2022-02-03

**Authors:** Kavita Jain, Darshita Sharma, Mala Patidar, Shirish Nandedkar, Ashish Pathak, Manju Purohit

**Affiliations:** 1 Department of Pathology, R.D. Gardi Medical College, Ujjain, India; 2 Department of Paediatrics, R.D. Gardi Medical College, Ujjain, India; 3 Department of Public Health Sciences, Karolinska Institutet, Stockholm, Sweden

**Keywords:** Sepsis, RDW, SOFA score, India

## Abstract

**Introduction:**

Early diagnosis of sepsis and its severity is essential for appropriate treatment to improve patient survival, especially in resource-limited settings. The aim of the present study was to study the role of red blood cell distribution (RDW) as a biomarker for the early detection of severe sepsis defined clinically and also in the prediction of mortality from sepsis.

**Methods:**

The cross-sectional study included a total of 175 subjects who met the inclusion criteria for the diagnosis of severe sepsis. After a thorough clinical examination, blood samples were taken from all patients within 3 hours of presenting the disease. The RDW values and other investigations were studied on the day of admission compared to other severity markers with the mortality index of 30 days.

**Result:**

The RDW value was significantly higher in patients with severe sepsis and in non-survivor patients than in survivors (*P* < .0001). There was a strong correlation between the SOFA score and RDW in predicting the disease outcome with the Pearson correlation coefficient of *r* = .46. The area under the receiver operating characteristic curve was found to be 0.852 at a CI of 95% (0.796-0.909) with RDW 17.15, sensitivity was 88.6% and specificity was 63.5%. There was a positive correlation with Pearson’s correlation coefficient of *r* = .46 between RDW and the SOFA score.

**Conclusions:**

RDW can be used as a potential marker for the early detection of severe sepsis and in the prediction of the outcome. Large multicenter prospective studies can confirm the utility of this routinely available marker for patients with sepsis.

## Introduction

Sepsis is a clinical syndrome of systemic inflammation that has severity from sepsis to septic shock and carries a high mortality rate and a high statistical burden.^1^ Data from the Center for Disease Control and Prevention, USA, reveal that sepsis is the leading cause of death in patients with noncoronary intensive care unit (ICU) and the tenth most common cause of death worldwide.^2^ The increase in mortality in patients diagnosed with sepsis can be attributed to multiple causes including advanced age patients, preexisting comorbidity, immunosuppressive diseases, therapies, or infections with multidrug resistant bacteria.^3^ Therefore, it is very important for clinicians to have the tools to identify and diagnose sepsis quickly, as early treatment can lead to an improvement in mortality and morbidity.

Several inflammatory biomarkers, clinical parameters and scoring systems have been used to assess the severity of sepsis and to predict mortality in patients with sepsis. Some of the common clinically used biomarkers and scoring systems include serum procalcitonin levels, serum C-Reactive Protein (CRP) and clinical scoring systems such as Sequential Organ Failure Assessment (SOFA), Quick SOFA (qSOFA), Acute Physiology and Chronic Health Evaluation (APACHE II) scoring systems. The degree of severity of sepsis is most often quantified by the SOFA score, which can predict the severity and outcome of multiple organ failure. However, calculating the SOFA score is cumbersome, especially in resource-constrained settings. In addition, the evaluation of the outcome of the septic patient during treatment should be focused, as the clinical and biological criteria currently used are undefined and inadequate for this purpose. The need for simple, cost-effective and easily available, yet reliable markers has pushed researchers to identify such markers to assess the severity and predict the prognosis of sepsis.

The Red Cell Distribution width (RDW) is one of the biomarkers that have been shown to predict the mortality and morbidity of sepsis.^4^ RDW is the coefficient of variation of the volume of red blood cells (RBC) and is a representation of the heterogeneity of the size of RBC (anisocytosis) of an individual patient.^5^ RDW is generally reported as part of the complete blood count (CBC) and is used in combination with the mean corpuscular volume to differentiate the cause of anemia.^6^ Studies have found important alterations in the shape of the red blood cells during the refractory phase of shock.^5,6^ They showed morphological and functional changes during sepsis in the RBC population and therefore alterations in RBC during shock and sepsis can contribute to multiple organ dysfunction syndrome. Many studies have reported that RDW is associated with a prognosis in critical illness, heart failure, acute myocardial infarction, pulmonary embolism, pneumonia, and cardiac arrest.^7-12^ Recently, most deaths in critically ill COVID-19 patients are caused by sepsis and RDW has been used as one of the biomarkers of outcome.^13^

In this study, the RDW hemogram parameter which is a part of the complete blood count, easy to evaluate and does not incur additional costs to routine analysis is studied to assess its efficacy as prognostic markers in sepsis and in predicting the clinical outcome as assessed by the SOFA score in patients with severe sepsis from rural tertiary settings.

## Methodology

The prospective cross-sectional study was conducted in the ICU of the Department of Medicine, and all laboratory investigations were carried out in the Department of Pathology, R.D. Gardi Medical College, Ujjain. The study was carried out from November 2018 to April 2020 among consecutive patients over 16 years of age identified as severe clinical sepsis.

The clinical criteria for sepsis were defined as: suspected or documented infection and an acute increase of ⩾2 SOFA points. Septic shock was defined as a subset of sepsis in which underlying abnormalities of circulatory and cellular metabolism are profound enough to substantially increase mortality. Septic shock was identified with a clinical construct of sepsis with persistent hypotension, which required vasopressor therapy to elevate MAP 65 mmHg despite adequate fluid resuscitation.^14^ Patients with febrile illness with clinical sepsis or in shock were examined and screened for evidence of SIRS criterion within 3 hours after admission. The patients were then enrolled in the study after obtaining the formal written informed consent of the patient or legal guardian.

DS recorded the details of demographic, clinical, provisional diagnosis, and laboratory parameters in a pre-designed and tested data collection form. The mean duration of stay was also observed for each patient and the clinical outcome was followed after discharge on phone call made 28 days from the day of admission. Blood samples were taken at the time of admission and sent for various laboratory parameters such as hemoglobin, platelet count, RDW, RBS, and serum electrolyte levels. RDW was measured as part of the CBC panel using an automated analyzer (Beckman Coulter Sysmex XN – 550). Patients who denied formal consent, were pregnant, had a history of blood transfusion in the previous week, known hematologic disorders, a history of bleeding, recent chemotherapy or had immunosuppression, were not willing to participate in the study or for investigations.

The patients were divided into 3 groups based on RDW at admission—as Grade I 14.5 (upper limit of the normal range of RDW), Grade II 14.6 to 17.3 and Grade III > 17.3 (Youden index, derived using the coordinates of the ROC curve of RDW with SOFA score). Severe sepsis was defined according to the Surviving Sepsis Campaign (SSC) guidelines updated in 2018^15^ and septic shock was considered when vasopressor was administered to patients to maintain mean arterial pressure of 65 mmHg or serum lactate value >2 mmol/L.^14^

Survivors were categorized as the patients who were alive, got cured, and were discharged from the hospital, whereas nonsurvivors were the patients who died during their course of treatment. Sepsis was suspected and severity scores were calculated using the values of clinical and laboratory parameters at the time of admission as the Quick Sequential Organ Failure Assessment (qSOFA) score, APACHE II, Sequential Organ Failure Assessment (SOFA) score, and Systemic Inflammatory Response Syndrome (SIRS) criteria.^16^ Ethical clearance was obtained from the Institutional Ethics Committee of R.D. Gardi Medical College, Ujjain.

The sample size was calculated based on prevalence with a confidence interval of 99% using the formula: n = z2*P* (100 − P)/d2 (*z* = 2.58 at a confidence interval of 99%, P = proportion in the target population = 51.5 % and d = degree of precision = 5%). Data were recorded in a questionnaire. Statistical analysis was performed using SPSS software version 23.0. All statistical tests performed were 2-tailed. 2*P* < .05 was considered statistically significant. Continuous data were expressed as mean ± standard deviation (SD) and interquartile range. Student’s *t*-test was used to analyze normal distributed continuous variables. Categorical variables were presented as percentages (%) and compared by means of the Chi-square test. An ANOVA test was performed to check for association between RDW and survivors and non-survivors. Correlation was done between the RDW and APACHE II and SOFA scores. The individual discriminatory values for sepsis of RDW, APACHE II and SOFA score were studied using receiver operating characteristic (ROC) curve analyses with calculation of area under the curve (AUC).

## Results

During the study period, a total of 175 patients were enrolled of which data was missing, with 6 patients and 3 patients denied for various investigations, thus data from 166 patients were finally analyzed. The mean age of the patients was 38.5 ± 27.5 years with a statistically significant difference (*P* < .0001) in the mean age (35.76 years) of survivors and (54.97 years) of non-survivors (Table 1). There was a slight female predominance 88/166 (53%).

**Table 1.** Clinical and laboratory parameters in survivors and non-survivors of sepsis patients (N = 166).

Variable	Survivor (N = 96)	Non-survivor (N =70)	*P*-value
Clinical parameters			
Age—y (mean ± SD)	35.7 ± 24.8	54.9 ± 20.9	.000
Gender (male)	46 (47.9)	32 (45.7)	.780
Diabetes mellitus	1 (1.0)	16 (22.8)	.000
Hypertension	1 (1.0)	10 (14.3)	.000
COPD and tuberculosis	0	8 (11.4)	.000
Others	0	10 (14.3)	.000
Laboratory parameters (mean ± SD)			
Hemoglobin—g/dL	9.7 ± 2.4	7.3 ± 1.6	.000
WBC count	28 544 ± 8716	29 406 ± 7722	.380
Platelet count—/µL	61 806 ± 33 370	56 481 ± 28 438	.270
RDW	16.4 ± 2.1	19.8 ± 2.6	.000
Duration of stay—d	7.8 ± 2.2	3.2 ± 1.7	.000
Severity score			
qSOFA, n (%)			
<2	19 (19.8)	1 (1.4)	.79
⩾2	77 (80.2)	69 (98.6)	
SOFA (mean ± SD)	6.9 ± 1.5	11.1 ± 1.7	.000
SIRS criteria, n (%)			
Tachycardia	86 (89.6)	70 (100.0)	.000
Tachypnea	77 (80.2)	69 (98.6)	.124
Leukocytosis/Leukopenia	75 (78.1)/6 (6.3)	43 (61.4)	.490/.820
Temperature > 38°C	93 (96.9)	59 (84.3)	.004

The most common clinical symptom was fever (91%), followed by shortness of breath (28.9%), diarrhea, and vomiting (27%). The mean systolic blood pressure was 70 ± 10.5 mmHg and the mean diastolic blood pressure was 40.8 ± 10.3 mmHg. Co-morbidities (diabetes mellitus, hypertension, chronic kidney disease, COPD, tuberculosis, and heart disease) were observed in 46/166 patients. A qSOFA score of 2 was observed in 146/166 (88%) patients, while the mean SOFA score was 8.7 ± 2.6 at admission. With SIRS criteria, tachycardia (94%) was the most common sign followed by hyperthermia (89.2%), tachypnea (88%), and leukocytosis (71.1%). The mean duration of hospital stay was 5.8 ± 3.0 days. Mean age, comorbidities, hemoglobin, mean RDW, stay duration, mean SOFA scores, tachycardia were significantly different (*P* < .05) between non-survivors and survivors (Table 1). Clinical symptoms such as fever, laboratory parameters such as mean hemoglobin concentration and serum Na^+^ levels, and mean stay duration were significantly (*P* = .000) lower in non-survivors compared to survivors. No statistical differences were observed in sex, clinical symptoms, WBC count, platelet count, RBS, serum K^+^ levels, and tachypnea between survivors and non-survivors. The mean RDW at admission in non-survivors (19.8 ± 2.6%) was significantly higher than that of survivors (16.4 ± 2.1%) (*P* = .000) (Table 1).

Grade I RDW was seen in 17 patients while Grade II and Grade III in 63 and 86 patients, respectively (Table 2). Patients with grade III RDW had significantly (*P* = .000) higher anemia proportion with mean hemoglobin of 7.1 ± 1.3 g/dL, and mean WBC count was significantly lower in patients with grade I compared to patients with grade II and grade III. However, no significant differences were observed in mean platelet count in all 3 groups. Patients with grade III RDW also had a significantly higher proportion of patients with low serum Na^+^ levels (136.0 ± 5.2 mmol/L) (*P* = .000). No statistical significance was observed in serum K^+^ and RBS levels. The mean SOFA score at admission was significantly higher in patients with RDW grade III (9.67) (*P* = .000). RDW was also found to have a significant graded association with SOFA score at admission showing a progressively increasing score along with an increasing RDW (*P* = .000). In the SIRS criteria, tachycardia, tachypnea, and leukocytosis were highly significant (*P* = .000). It was also seen that co-morbid conditions were more common in patients with grade III RDW and the mean stay duration (1.31 days) was shorter in them, although both are not significantly different from other groups.

**Table 2.** Comparison of clinical and laboratory parameters in sepsis patients presented with various grades of RDW.

Variables	Grade I* (n = 17)	Grade II* (n = 63)	Grade III* (n = 86)	*P* value
Demographic and clinical parameters, n (%)				
Gender (male)	9 (52.9)	26 (41.3)	43 (50.0)	.501
Male:female ratio	1:1.1	1:0.96	1:1	-
Age, y (range)	17 (3 d-80 y)	63 (0 d-82 y)	86 (9 d-87 y)	.593
Diabetes mellitus	0	3 (4.8)	14 (16.3)	.02
Fever with chills	16 (94.1)	59 (93.6)	76 (88.4)	.000
Unconsciousness	1 (5.9)	3 (4.8)	20 (23.3)	.004
Hypertension	0	1 (1.6)	10 (11.6)	.03
COPD and tuberculosis	0	2 (3.2)	6 (6.9)	.74
Others	0	2 (3.2)	8 (9.3)	.41
Laboratory parameters				
Hemoglobin —g/dL (mean ± SD)	12.5 ± 2.0	9.8 ± 1.9	7.1 ± 1.3	.000
MCV (fL)	84.1 ± 13.3	84.8 ± 11.9	83.5 ± 15.1	.040
MCH (pg)	30.1 ± 3.6	30.6 ± 4.2	29.5 ± 5.5	.005
MCHC (g/dL)	34.4 ± 0.9	34.2 ± 1.3	34.4 ± 1.3	.538
RBS (mean ± SD)	163.4 ± 30.1	159.0 ± 42.8	161.3 ± 36.3	.591
Na^+/^K^+^ (mean ± SD)	141.1 ± 1.5/4.4 ± 0.3	140 ± 2.5/4.2 ± 0.4	136 ± 5.2/4.4 ± 0.5	.000/.161
Severity score				
qSOFA, n (range)	2 (1-2)	2 (1-2)	2 (1-2)	.000
SOFA, n (range)	7 (6-12)	8 (5-12)	10 (4-14)	.000
SIRS criteria				
Tachycardia (beats/min)	7 (41.2)	63 (100.0)	86 (100.0)	.000
Tachypnea (breaths/min)	10 (58.8)	57 (90.5)	79 (91.9)	.000
TLC ⩾ 12 000 cells/mm^3^	3 (17.6)	52 (82.5)	63 (73.2)	.000
TLC ⩽ 4000 cells/mm^3^	1 (5.9)	8 (12.7)	23 (26.7)	.000
Temperature > 38°C	16 (94.1)	59 (93.6)	73 (84.9)	.185

*Grade I—RDW ⩽ 14.5; Grade II—RDW 14.6-17.3; Grade III—RDW > 17.3.

**Figure 1.**
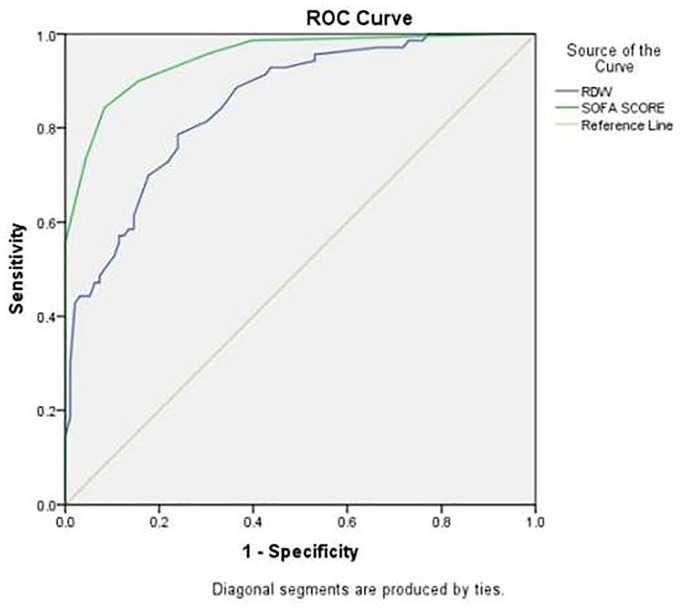
ROC curve showing relationship between RDW and SOFA score.

As is evident from Table 3 the maximum AUC (0.98) was obtained for heart rate (95% confidence interval [CI], 0.959-0.999), (*P* < .001). The AUC for the SOFA score was 0.95 (95% CI, 0.918-0.981) (*P* < .001) (Figure 1). Table 3 also shows the sensitivity and specificity of various parameters at different RDW cutoffs. However, in multivariate logistic regression analyzes, the SOFA score at admission, the qSOFA score, heart rate, respiratory rate, total leukocyte count and RDW were found to be independent predictors of the outcome of patients with severe sepsis patients (*P* < .05) (Table 4).

**Table 3.** Sensitivity and specificity of laboratory parameters in severe sepsis patients (N = 166).

Parameter	RDW cut off	AUC	Std. error	95% CI	*P* value	Sensitivity, %	Specificity, %
SOFA score	17.3	0.95	0.016	0.918-0.981	.000	72.9	95.8
Hb	17.4	0.86	0.028	0.809-0.918	.000	68.8	100.0
HR	16.1	0.98	0.010	0.959-0.999	.000	85.9	100.0
RR	17.1	0.70	0.071	0.557-0.836	.004	64.4	65.0
TLC	15.1	0.72	0.046	0.626-0.805	.000	96.6	35.4
Platelet	16.1	0.55	0.120	0.317-0.790	.685	81.4	40.0
Na^+^	18.1	0.91	0.023	0.868-0.958	.000	87.8	75.2
RBS	15.1	0.44	0.060	0.322-0.558	.361	96.0	12.1

**Table 4.** Multivariate logistic regression analysis for the outcome in study population of 166 patients with severe sepsis.

Variable	Regression coefficient	95 % CI	*P* value
SOFA	0.11	0.088 to 0.125	.000
qSOFA	0.21	0.070 to 0.346	.003
HR	0.00	0.002 to 0.006	.000
RR	−0.01	−0.011 to −0.003	.000
TLC	−0.00	−0.000 to −0.000	.002
RDW	0.03	0.005 to 0.048	.014

## Discussion

The study reveals that RDW was significantly different in patients with severe sepsis and survivors and thus can be used as a potential marker for early detection of severe sepsis and in predicting the outcome of sepsis. There is a good correlation of the SOFA score with RDW and there is a significant increase in RDW in patients with severe sepsis. It is a quick, easy, and nonexpensive predictor in emergency resource-constrained settings.

In our study a gradual increase and a positive correlation of RDW with increasing age are observed; however, no significant gender difference was observed in RDW values. In a study^17^ of 3226 participants, high RDW was significantly detected in patients with >50 years of age (mean 57 years; *P* < .001), Similarly others^18^ found that among 26 820 participants of > 45 years of age (62% women), high RDW was significantly observed in older patients (mean 59 years; *P* < .001) patients with no significant gender differences.

In our study a graded association was found between RDW and the SOFA score with high statistical significance (*P* = .000), being 10 (4-14) with RDW >17.3, 8 (5-12) with RDW 14.6 to 17.3, and 7 (6-12) with RDW <14.5. The level of RDW has been shown to correlate with the SOFA score suggesting a parallel increase with the severity of the disease and is an index of multiple organ dysfunction in sepsis. It is suggested that the presence of inflammatory cytokines causes dysregulated erythropoiesis that is reflected in increased RDW.^19^ It is also seen that sepsis oxidative stress decreases the life span of RBCs, thus releasing new RBCs that lead to increased RDW.^20^

The mean RDW was significantly (*P* < .0001) elevated in non-survivors (19.81%) compared to survivors (16.43%) of severe sepsis and mortality was significantly (*P* < .0001) elevated among patients with severe sepsis with increased RDW. Non-survivors had a high SOFA score (11.1 ± 1.7). This is an important finding to be noted. A high statistical significance and a positive correlation was obtained between the SOFA score and the outcome (*P* < .0001), where on admission the SOFA score in survivors was significantly lower than the SOFA score in non-survivors (*P* = .005).^21^ It has recently been reported that in addition to many other changes leading to microcirculatory changes in sepsis, RDW > 15 affects the deformability of RBCs.^22^ Deformed RBCs further increase the activation of the immune response of phagocyte cells leading to organ dysfunction in sepsis.^22^

The diagnostic accuracy of outcome prediction, RDW showed a fair area under the ROC curve 0.852, CI of 95% (0.796-0.909). The AUC between the RDW and SOFA score with a reference curve was 0.852, CI 95% (0.796-0.909) and 0.950, CI 95% (0.918-0.981). A positive correlation with Pearson’s correlation coefficient of *r* = .46 between RDW and SOFA score indicates that an increase in SOFA score is directly related to an increase in RDW levels. Mortality rates increased when the RDW value was high; therefore, RDW can be used as a prognostic marker in severe sepsis. In multivariate logistic regression analyzes, the SOFA score at admission, qSOFA, heart rate, respiratory rate, and total leukocyte count were found to be independent predictors of severe sepsis (*P* < .05).

Various laboratory parameters were also compared among survivors and non-survivors. We found that in non-survivors, hemoglobin was low (7.3 ± 1.6), ESR was high (76.0 ± 24.8), platelet count was low (56 481 ± 28 438), RDW was high (19.7 ± 2.6), serum Na^+^ levels were low (135.7 ± 5.4) and total bilirubin was high (3.6 ± 1.1) compared to survivors. We found that hemoglobin, Na^+^, and bilirubin were highly significant indicating their role in severe sepsis, while no significant changes were shown with the MCV, MCH, MCHC, RBS, serum K^+^, SGOT, SGPT, ALP and albumin/globulin ratio. Critically ill patients with sepsis generally have high hemoglobin concentrations and are associated with a higher risk of death as Hbβ is increased in severe sepsis and may represent a novel marker of endothelial cell dysfunction.^23^ Although hemoglobin increased significantly, ESR has only little significance in severe sepsis.^24^

There are certain limitations of the study. Since RDW is affected by many conditions, RDW without other inflammatory indicators such as C-reactive protein and gamma-glutamyl transferase may not provide exact information on the patient’s inflammatory status. In addition, the underlying diseases of the patient could alter the RDW levels. This was a prospective observational study in a single institution in a short period with a smaller sample size, and a single baseline RDW was observed compared to serial changes in RDW over the period of illness. For validation of the results, the sample size should be large. The time elapsed between blood sampling and measurement of RDW may significantly affect RDW levels, however, in the present study all RDW measurements were performed within 4 hours of blood collection. All these necessitate further clinical research for future prospective multicenter and randomized trials to evaluate prognostic role of this simple marker with reducing most of the possible biases.

In conclusion, our data raise the promising role of RDW measurement as an easily available, simple, robust, and inexpensive potential marker in the emergency for early prediction of the severity and outcome of sepsis patients in resource-strained settings where tertiary care facilities such as arterial blood measurement are not available. There was a good correlation of the SOFA score with the RDW in predicting the outcome. Larger studies are essential before extrapolating these data.
